# Biofunctionalization of Xenogeneic Collagen Membranes with Autologous Platelet Concentrate—Influence on Rehydration Protocol and Angiogenesis

**DOI:** 10.3390/biomedicines10030706

**Published:** 2022-03-18

**Authors:** Sebastian Blatt, Saskia-Vanessa Schröger, Andreas Pabst, Peer W. Kämmerer, Keyvan Sagheb, Bilal Al-Nawas

**Affiliations:** 1 Department of Oral and Maxillofacial Surgery—Plastic Operations, University Medical Center Mainz, Augustusplatz 2, 55131 Mainz, Germany; saskia.schroeger@unimedizin-mainz.de (S.-V.S.); peer.kaemmerer@unimedizin-mainz.de (P.W.K.); keyvan.sagheb@unimedizin-mainz.de (K.S.); al-nawas@uni-mainz.de (B.A.-N.); 2 Department of Oral and Maxillofacial Surgery, Federal Armed Forces Hospital, Rübenacherstr. 170, 56072 Koblenz, Germany; andreas.pabst@uni-mainz.de

**Keywords:** platelet-rich fibrin, collagen matrix, in ovo yolk sac, angiogenesis, pH value, guided bone regeneration

## Abstract

Background: The aim of this study was to analyze possible interactions of different xenogeneic collagen membranes (CM) and platelet-rich fibrin (PRF). PH values were evaluated in the CM rehydration process with PRF, and their influence on angiogenesis was analyzed in vivo. Materials and Methods: Porcine (Bio-Gide^®^, Geistlich)- and bovine-derived collagen membranes (Symbios^®^, Dentsply Sirona) were biofunctionalized with PRF by plotting process. PRF in comparison to blood, saline and a puffer pH7 solution was analysed for pH-value changes in CM rehydration process in vitro. The yolk sac membrane (YSM) model was used to investigate pro-angiogenic effects of the combination of PRF and the respective CM in comparison to native pendant by vessel in-growth and branching points after 24, 48 and 72 h evaluated light-microscopically and by immunohistochemical staining (CD105, αSMA) in vivo. Results: Significantly higher pH values were found at all points in time in PRF alone and its combined variants with Bio-Gide^®^ and Symbios^®^ compared with pure native saline solution and pH 7 solution, as well as saline with Symbios^®^ and Bio-Gide^®^ (each *p* < 0.01). In the YSM, vessel number and branching points showed no significant differences at 24 and 48 h between all groups (each *p* > 0.05). For PRF alone, a significantly increased vessel number and branching points between 24 and 48 h (each *p* < 0.05) and between 24 and 72 h (each *p* < 0.05) was shown. After 72 h, CM in combination with PRF induced a statistically significant addition to vessels and branching points in comparison with native YSM (*p* < 0.01) but not vs. its native pendants (*p* > 0.05). Summary: PRF represents a promising alternative for CM rehydration to enhance CM vascularization.

## 1. Introduction

The demand of further development of tissue regeneration techniques and materials in oral regeneration has been increased continuously [1]. In general, components used in tissue regeneration, such as stem cells, cytokines, and growth factors, are embedded in 3D (three-dimensional) structures in an attempt to replicate and restore the injured soft and hard tissues [2]. Since their introduction, resorbable, naturally derived collagen membranes (CM) of xenogeneic origin have been widely used in guided bone and tissue regeneration (GBR/GTR) procedures. The proven biocompatibility and ability of CM to promote wound healing has been cited as a major advantage [3]. In recent approaches, CM were successfully used as tissue-engineered 3D structures (e.g., tissue grafts, biomaterials, cell-assembled tissue equivalents) that have the capacity to deliver in a localized and sustained manner viable cell populations and/or bioactive/therapeutic molecules for regeneration processes [4]. However, the possible lack of or delayed blood vessel supply after implantation can lead to insufficient oxygen and nutrient supply, and potentially to necrosis and failure of the equivalent that remains a significant clinical limitation [2]. A key challenge is the establishment of a rapid and sufficient vascularization for tissue constructs, guaranteeing long-term survival and function [5]. Here, pro-angiogenic features are of special interest as a synergism of physical-chemical stimuli to enhance angiogenesis and therefore local blood supply that initially delivers oxygen and all other nutrients to fuel osteogenesis simultaneously [6]. However, translation of these approaches into a clinical workflow is limited, mainly due to restrictive reasons. As a clinical method to emphasize pro-angiogenic features of the respective biomaterials, autologous platelet concentrates (PC) are in special focus of different research approaches [7]. Platelets contain high quantities of key growth factors, such as vascular endothelial growth factor (VEGF), that stimulates relevant cell features, such as proliferation, differentiation and migration [8]. This leads to the recruitment of incoming regenerative cells in the defective locations, not only after clinical procedures such as tooth extractions but also for tissue engineering methods [9,10]. Furthermore, growth factors play a key function in the intravascular innate immune system [11] originating from fibrin glues firstly described 40 years ago for wound healing. In the first generation, PC blood is collected with anticoagulants during surgery and immediately processed by centrifugation to separate the blood into red blood cells, acellular plasma and the ‘buffy coat’ layer in between, in which platelets are concentrated. Depending on different protocols, this phase is applied to the surgical site with a syringe, together with thrombin and/or calcium chloride (or similar factors) to trigger platelet activation and fibrin polymerization [8]. As a further development, second generation PC, such as platelet-rich fibrin (PRF), are manufactured without any anticoagulants or biochemical modification of the blood and are therefore subject to a natural coagulation process that allows subsequent release kinetics of growth factors in accordance with natural wound healing phases [12]. In this process, the dense fibrin network acts as a reservoir for tissue growth factors at injured sites [13]. Furthermore, the presence of leukocytes in second generation PCs appears to play an important role in wound healing [14], that may be used inter alia for regeneration of periodontal intrabony defects [15] and gingival tissue regeneration [16]. The combination of PRF and CM seems promising for clinical application [17,18]. Here, especially in maxillary sinus augmentation and dental implant restorative procedures, the combination of PRF and CM did show positive results [19]. It was found that the degree of porosity, hydrophilic nature and surface polarity seem to be major factors in the absorption of the liquid PRF [20]. In addition, Transforming Growth Factor β (TGF-β) directly adsorbs to CM [21]. In a recent study that focused on initial interaction between PRF and CM, it was demonstrated that the combination of PRF and three different porcine CM led to a significantly increased growth factor release in vitro after 24 h for all PRF-activated CM in comparison with native CM at a similar level to PRF alone. Furthermore, a significantly increased angiogenic potential was seen in vivo after 24 h [22]. Beside the early pro-angiogenic benefits of the combination of PRF and CM, PRF incubation could also serve as a method to adequately rehydrate the respective CM before its clinical use. Rehydration is recommended by the manufacturers, but regarding rehydration, both the medium and period are often vague with a lack of evidence. It was demonstrated that the rehydration protocol can significantly affect the biomechanical properties of CM [23]. So far, it does not seem to be elucidated if changes in pH value occur depending on chosen incubation medium, such as saline solution, blood, or autologous platelet concentrates, for the CM during rehydration protocol, e.g., by leaching chemical residuals of the processing procedures. As acidity/alkalinity can influence wound healing via regulation of angiogenesis, the formation of collagen and the cellular activity of macrophages [24], and combination of CM with PRF could not alone effect pro-angiogenic features directly, but also influence blood vessel formation indirectly during rehydration process when used for CM incubation. In this context, there is rising evidence that pH levels directly influence neovascularization [25]. The aim of this study was to analyze possible changes in pH values in CM rehydration with PRF and to compare the combination of PRF with CM of bovine and porcine origin concerning the pro-angiogenic potential at different times in vivo.

## 2. Materials and Methods

### 2.1. Collagen Membranes

Bio-Gide^®^ (Geistlich Biomaterials Vertriebsgesellschaft mbH, Baden-Baden, Germany) is a porcine-derived collagen matrix (CM) without any artificial cross-linking. It has a native bilayered structure derived from porcine peritoneum with a good liquid uptake that provides fibroblast and osteoblast proliferation within its porous structure [26,27]. Symbios^®^ (Dentsply Sirona Deutschland GmbH, Bensheim, Germany) is a bovine-derived, nonfriable CM that contains highly purified type I collagen fibres. All CM were prepared in a size of 4 × 4 mm (±0.1 mm) by using a sterile scalpel. The parameters were controlled by a sterile caliper. All procedures were performed under sterile conditions.

### 2.2. Platelet-Rich Fibrin Protocol

To produce platelet-rich fibrin (PRF), venous blood was collected from two healthy volunteers who gave informed consent as previously described [28]. Briefly, special vacutainer systems (A- and i-PRF+; Process for PRF, Nice, France) with a volume of 10 mL were used, and the tubes were placed immediately in the centrifuge according to the protocol (1200 rpm for 8 min, relative centrifugal force 177× *g* at a fixed angle rotor with a radius of 110 mm; Duo centrifuge, Process for PRF, Nice, France). The stable PRF was pressed manually with its appropriate PRF-box (Process for PRF, Nice, France) for 60 s and cut into pieces of 4 × 4 mm in size by using a sterile scalpel under sterile conditions. For biofunctionalization, Bio-Gide^®^ and Symbios^®^ CM were pressed together with the PRF scaffolds. The following samples were analyzed: native Bio-Gide^®^ matrix (BM), native Symbios^®^ matrix (SM), combination of PRF and Bio-Gide^®^ matrix (BBI), combination of PRF and Symbios^®^ matrix (SBI), and PRF alone. All procedures were conducted in accordance with the Declaration of Helsinki and approved by the Ethics Committee of Landesärztekammer Rhineland-Palatine (no. 2019-14705_1).

### 2.3. Measurements of Differences in pH Value during Rehydration

To test changes in pH value during the rehydration of the CM, samples were incubated for 20 min with the following media (5 mL): venous blood, buffer solution with a fixed pH value of 7, saline solution, and PRF. The following groups were analyzed: native saline, saline with Bio-Gide^®^ matrix, saline with Symbios^®^ matrix, native pH 7 solution, pH 7 solution with Bio-Gide^®^ matrix, pH 7 solution with Symbios^®^ matrix, native PRF, PRF with Bio-Gide^®^ matrix, PRF with Symbios^®^ matrix, native blood, blood with Bio-Gide^®^ matrix, blood with Symbios^®^ matrix. The pH value was measured every 5 min with a glass pH electrode (pH-Meter CG840; Schott, Mainz, Germany). To avoid clotting of the venous blood, 2 mL of heparin was added (25,000 IU/5 mL solution; LEO Pharma GmbH, Neu-Isenburg, Germany). Before every testing, calibration with buffer solutions of pH value 4 and 7 was performed. The experiment was repeated nine times per CM and media.

### 2.4. Yolk Sac Membrane Assay for Vessel and Branching Point Quantification

**Figure 1:**
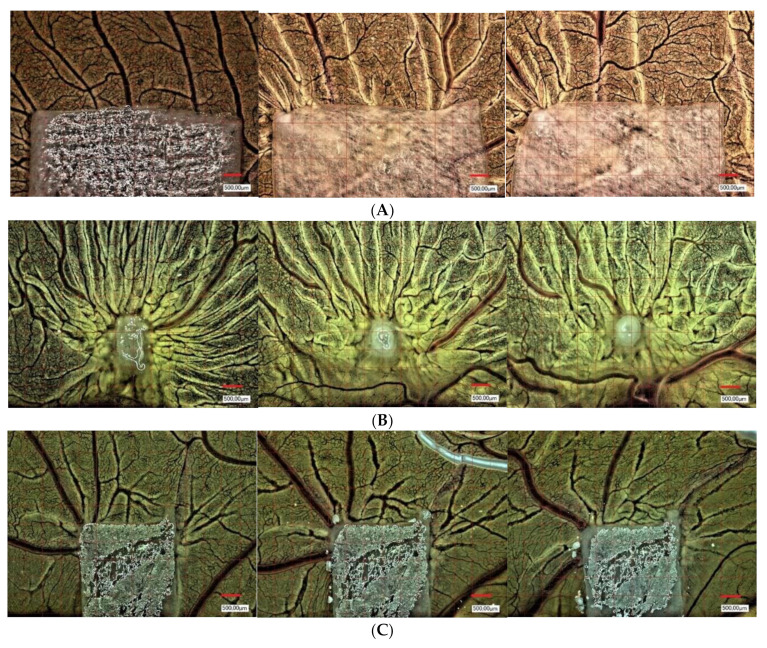
Example of microscopic analysis. (**A**) Bio-Gide^®^ matrix, (**B**) PRF matrix, (**C**) Symbios^®^ matrix after 24, 48 and 72 h in the YSM assay. Magnification: 50-fold.

**Figure 2:**
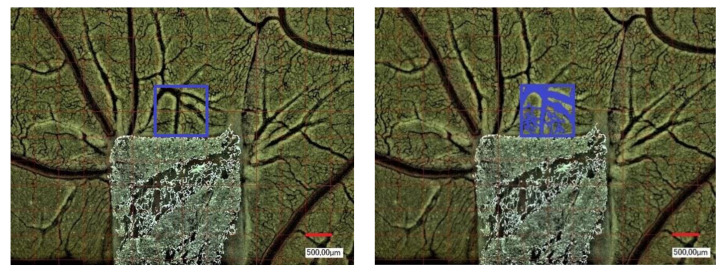
Example of microscopic analysis of vessel and branching points. After centering the matrix, the same region of interest (ROI) of 500 × 500 µm was standardly applied for every experiment. The number of vessels and the branching points (highlighted in blue) per mm² of the ROI were counted manually. Magnification: 50-fold.

Pro-angiogenic properties of the respective CM (native and in combination with PRF) in comparison to PRF alone and a negative yolk sac membrane (YSM) control were evaluated in vivo (Figure 1). An experimental, standardized protocol was designed for each series of tests as previously described [22]. Briefly, fertilized Leghorn chicken eggs (LSL Rhein-Main, Dieburg, Germany) were first cleaned, marked at the pointed pole, and numbered. They were incubated in a special incubator (Janeschitz, Hammelburg, Germany) at a temperature of 37.5 °C and constant humidity. Three days after incubation, eggs were cleaned again, and a transparent adhesive strip was attached to the marked poles. With a single syringe, 8–10 mL of egg clear was collected from the marked pole. Afterwards, the pole was closed again with transparent adhesive tape to avoid contamination. On the upper surface, an oval 3 × 3 cm opening into the surface was cut with sterile scissors. For further incubation, the whole was closed with parafilm. The following day, CM to be tested were cut into approximately 4 × 4 mm pieces and inserted under sterile conditions onto the YSM, embryo-distant, near the vessels. The YSM alone was used as negative control group (native) and the eggs further sealed with parafilm and incubated as described above. At subsequent time points after 24, 48, and 72 h, the vascularization near the CM was photo-documented by centering the middle of the CM with a digital microscope at 50- and 100-fold magnification (VHX-1000; Keyence, Neu-Isenburg, Germany, Figure 1). The same region of interest (ROI) of 500 × 500 µm was standardly applied for every experiment (*n* = 9 per respective CM, in total *n* = 135), the number of vessels and the branching points per mm² of the ROI were analyzed with the corresponding software (CV-H1X Software; Keyence, Neu-Isenburg, Germany, Figure 2). CM were removed together with the YSM under sterile conditions after 24, 48 and 72 h, placed on weighing paper and positioned in an embedding cassette in Roti-Histofix 4.5% for further histological preparation. Care was taken to ensure that the embryos were euthanized quickly by the separation of the main vessels after the experiments.

### 2.5. Histological Preparation

The tissue fixed in Roti-Histofix 4.5% was cut according to the standard instructions for paraffin embedding, placed in embedding capsules, and transferred to the single-embeds machine. For further histological processing and archiving, the samples were cast, placed in the right cutting direction, and cooled. The blocks were then cut into 5 µm-thick slices using a HistoCare-autocut (Leica Biosystems, Wetzlar, Germany). They were first inserted into a cold water bath made of aquadest and then into a 40 °C hot water bath to unfold. The samples (*n* = 9 per respective CM, in total *n* = 135) were transferred to a slide and prepared for staining and drying. For immunohistochemical stains, coated slides were used, and uncoated slides were used for the hematoxylin-eosin (H&E) stains. Before the actual staining, all histological samples were deparaffined and placed 3 × 15 min in xylene.

### 2.6. Hematoxylin-Eosin Staining

For H&E-staining (Merck, Darmstadt, Germany) the samples were placed 5 min in haematoxylin (1:10 diluted), then 10 min under running tap water and 1–2 min in eosin as previously described [11]. After a short time in aquadest, the slides were briefly swung in 70% alcohol and in 96% alcohol. Afterwards, they were placed in 100% alcohol for at least 5 min and in xylene for 5–10 min. With the help of thin cover glasses and eukitt, the cuts were covered and sealed. In the end, nuclei were stained blue and cell plasma was stained red.

### 2.7. Anti-Alpha Smooth Muscle Actin Antibody(1A4) (α-SMA-Staining)

For α-SMA staining the antibody A2547 mouse (1:1000; Sigma-Aldrich, St. Louis, MO, USA) was used [11]. The samples were inserted in a descending range of alcohol: 2 × 100%, 96%, 70% and 50% ethanol, for 5–10 min each. Then, they were placed in phosphate-buffered salt solution (PBS; Sigma-Aldrich, St. Louis, MO, USA) for 20 min. For unmasking, the slides were inserted 20–40 min into the steam pot, which was filled with sodium-citrate buffer of a pH value of 6. All cuts were washed 10 min before the peroxidase block (Dako, Jena, Germany) was applied for 5 min. After a further 10 min, washing using PBS and the Dako protein block (Dako, Jena, Germany) was added for 10 min. The primary antibody was applied and after 1 h incubation, marked polymer-HRP anti mouse (Dako, Jena, Germany) was added on the cuts for 30 min. After 10 min of washing with PBS, a DAB substrate was applied to the slides for up to 10 min. After 5 min in haematoxylin, running water was poured over them for 5 min and the slides were treated in an ascending range of alcohol (70%, 96%, 100%). They were placed in xylene for 5 min, covered with eukitt and sealed with thin cover glasses.

### 2.8. Immunofluorescence CD105 Anti-Chicken Antibody

For CD105 staining (Biorbyt, Cambridge, England) samples were placed in in a descending range of alcohol: 2 × 100%, 96%, 70% and 50% ethanol, for 5–10 min. They were treated for 20 min with phosphate buffered salt solution (PBS). According to protocol [22], the following steps were performed: 3 × 5 min washing in PBS, 5 min adding of Triton-X-100 0.1% (10 mL PBS + 10 µL Triton-X-100; Sigma-Aldrich, St. Louis, MO, USA), 2 × 5 min PBS. Blocking was performed 5 min with PBS/BSA 5% (Sigma-Aldrich, St. Louis, MO, USA), PBS/Goat NS block (Dako, Jena, Germany) and 5 min PBS afterwards. CD105 anti-chicken antibody was added for 1 h (diluted 1:750 with PBS/BSA 1%), 3 × 5 min PBS and 60 min a second antibody alpha-rabbit 488 (diluted 1:100 with PBS/BSA 1%; Invitrogen, Carlsbad, CA, USA). After another 3 × 5 min in PBS cell nucleus staining was performed with DAPI (1:1000; ThermoFischer, Waltham, MA, USA) covered with fluorescence mount medium (Dako S3023; Dako, Jena, Germany).

### 2.9. Microscopic Analysis

**Figure 3:**
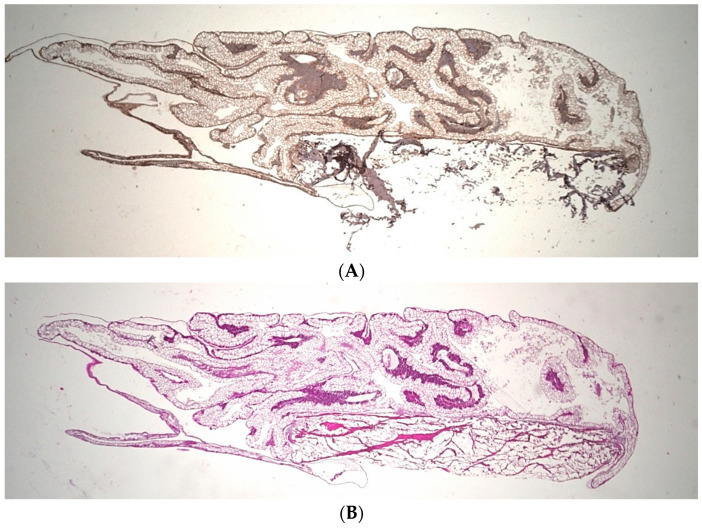

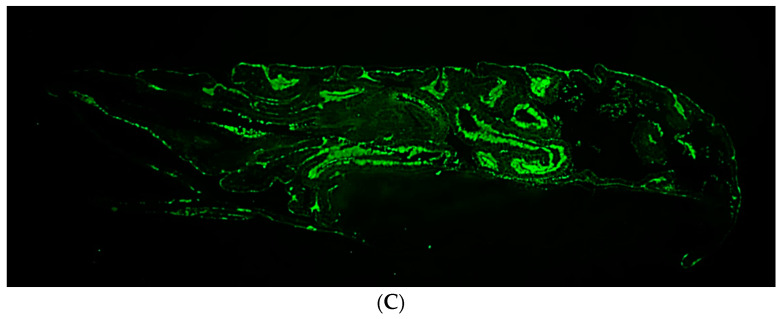
Example of immunohistochemical staining, (**A**) aSMA, (**B**) HE, (**C**) CD 105. Magnification: 2-fold.

The resulting stained specimens were examined and photo-documented using the Keyence Biorevo BZ-9000 microscope (Keyence, Neu-Isenburg, Germany) and its corresponding BZII-Viewer-Analyzer program (Brightfield HF and Phako, microscope position 2 Plan Apo Na.10; Keyence, Neu-Isenburg, Germany) as previously described (Figure 3) [22]. The pictures were analyzed using the application Hybrid-Cell-Count and Brightfield & Single extraction. With the subsequent adjustment of tolerance and transparency, the ratio of strongly stained sections of histological tissue to less stained areas could be calculated as a portion (in percentage) of the whole sample.

### 2.10. Statistical Analysis

For statistical analysis, SPSS (version 27; IBM, Ehningen, Germany) was used. Initially, a Kolmogorov–Smirnov and Shapiro–Wilk test were applied to verify the condition for parametric tests. The graphic representation of histograms, boxplots, Q-Q plots, skewness, and kurtosis was also examined. After all parameters showed no normal distribution, further analysis was carried out using Kruskal–Wallis test to identify significant differences between the different CM, but also to identify differences within the individual groups in time. If differences could be verified, pairwise comparisons were carried out using single-factor ANOVA according to Kruskal–Wallis. For pH value analysis, the Friedman test was used to identify differences within the measurements within 20 min. Differences between the CM were also verified with a single-factor ANOVA, according to the Kruskal–Wallis test. Boxplots were used for data illustration. *p*-values *p* < 0.05 were considered statistically significant.

## 3. Results

### 3.1. pH Value Analysis

**Figure 4:**
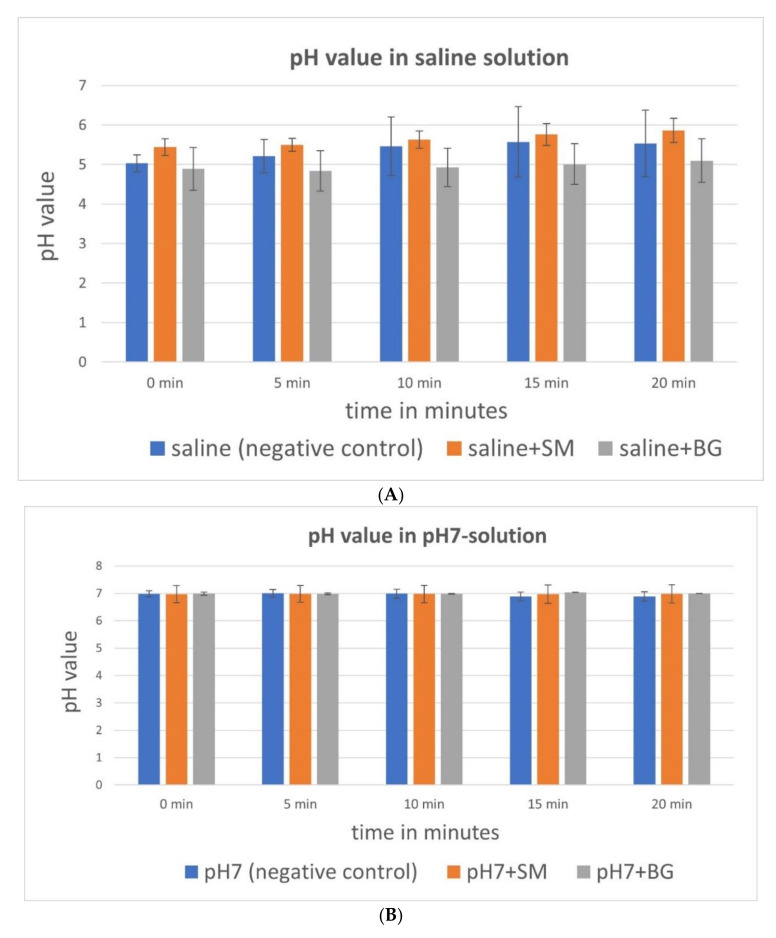

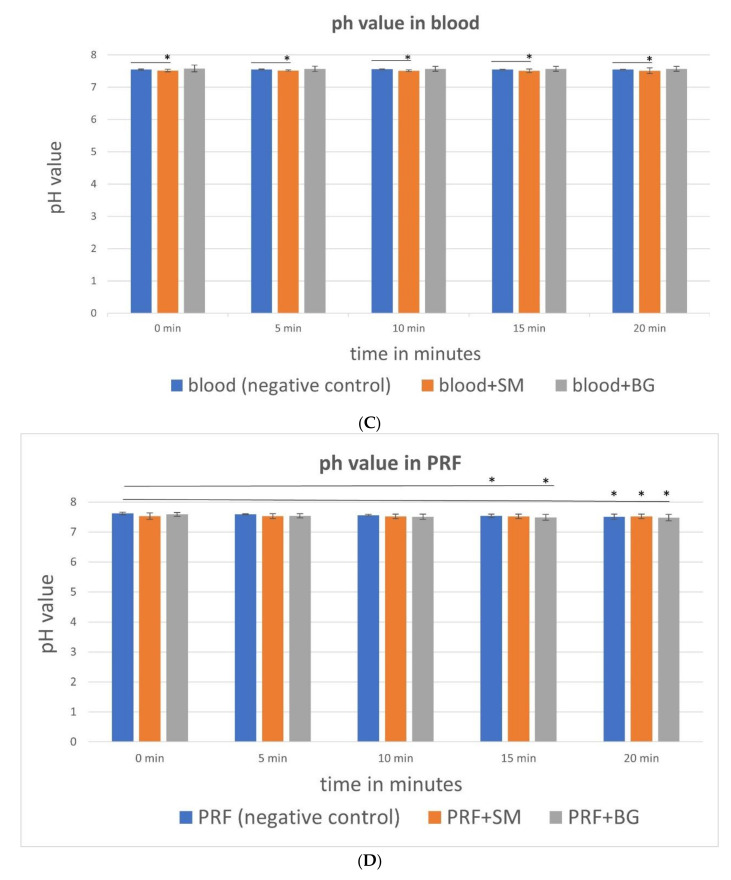
Differences in pH value during 20 min of rehydration of the respective CM depending on the media: (**A**) saline solution, (**B**) ph 7 solution, (**C**) blood, (**D**) PRF. Blue column: negative control media, orange column: media plus Symbios^®^ (_S), grey column: media plus Bio-Gide^®^ (_B). * marks statistically significant differences (*p* < 0.05; for details, please see the responding paragraph).

The average pH value after 20 min of incubation of the analysed media were in saline solution 4.95 (±0.451 SD), in pH 7 solution 6.98 (±0.042 SD), in blood 7.52 (±0.082 SD) and in PRF 7.76 (±0.115 SD). The pH values in the saline and pH 7 solutions stayed constant, even after inserting the different membranes and for all different time points. PRF group displayed a significant decrease in the measured pH value during the observation time of 20 min (Symbios^®^: *p* < 0.05, Bio-Gide^®^: PRF alone: *p* < 0.001). Blood combined with Symbios^®^ membrane also showed a significant difference; a lesser pH value was shown than in the native form (*p* < 0.01). Significantly higher values were found at all points in time in PRF and its combined variants with Bio-Gide^®^ and Symbios^®^ compared with native saline solution, as well as saline with Symbios^®^ and Bio-Gide^®^ (each *p* < 0.01). Blood also showed higher pH values over time in comparison with saline solution (*p* < 0.05). In addition, PRF achieved higher pH values over time compared with the pH 7 solution (*p* < 0.05, Figure 4).

### 3.2. Yolk Sac Membrane Assay

**Figure 5:**
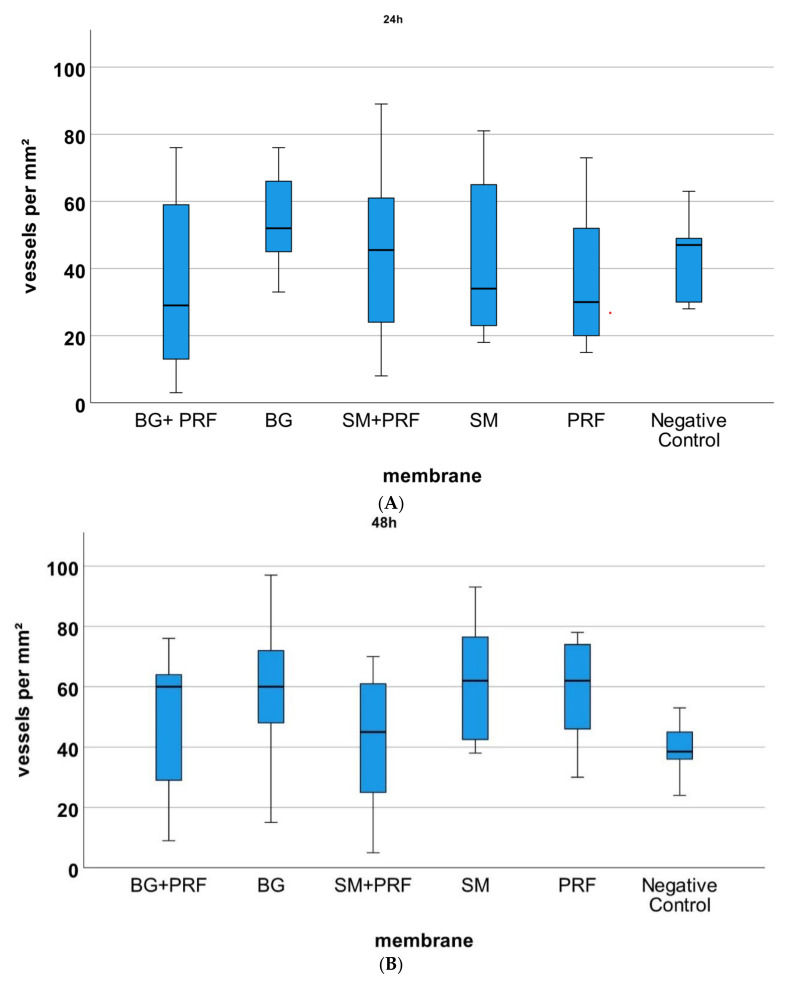

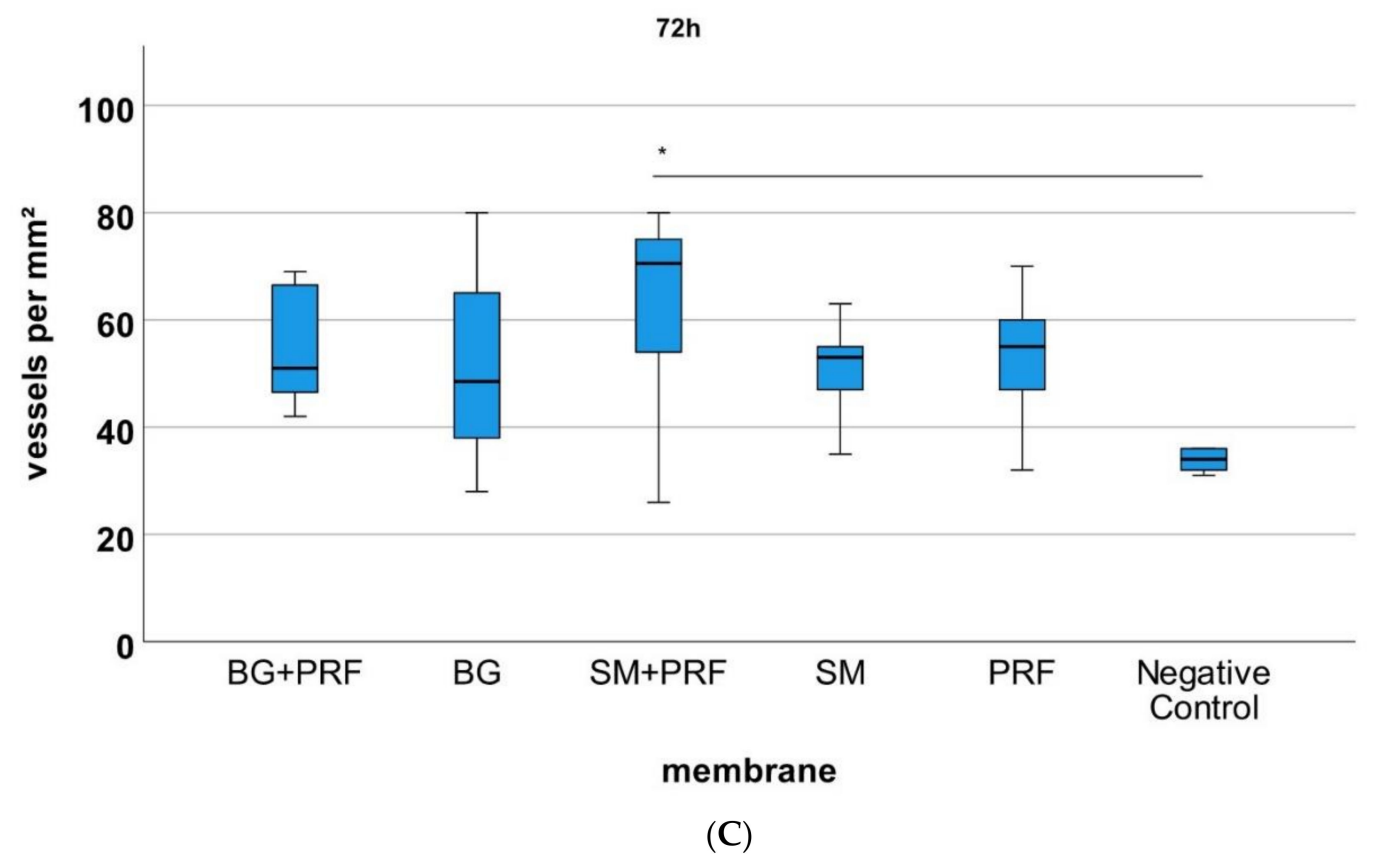
YSM assay and analysis of the vessels per mm^2^ for the respective matrix (BG + PRF: Bio-Gide^®^ with PRF, BG: native Bio-Gide^®^, SM + PRF: Symbios^®^ with PRF, SM: native Symbios^®^), PRF negative control. (**A**) 24 h, (**B**) 48 h, (**C**) 72 h of incubation. * marks statistically significant differences (*p* < 0.05; for details, please see the responding paragraph).

**Figure 6:**
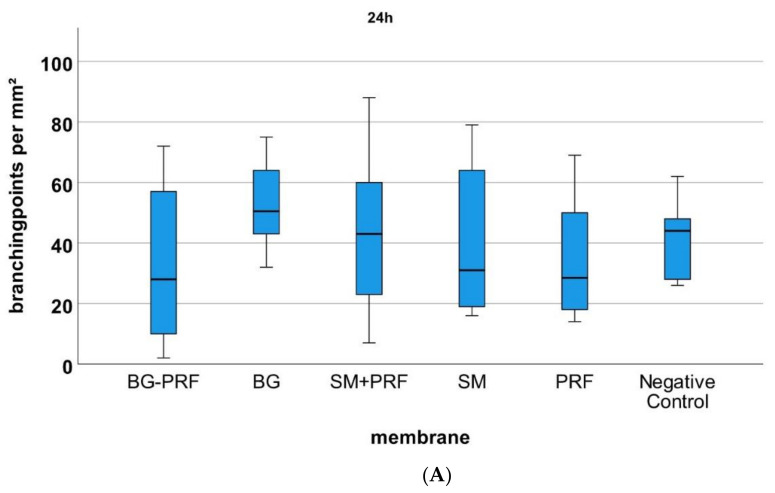

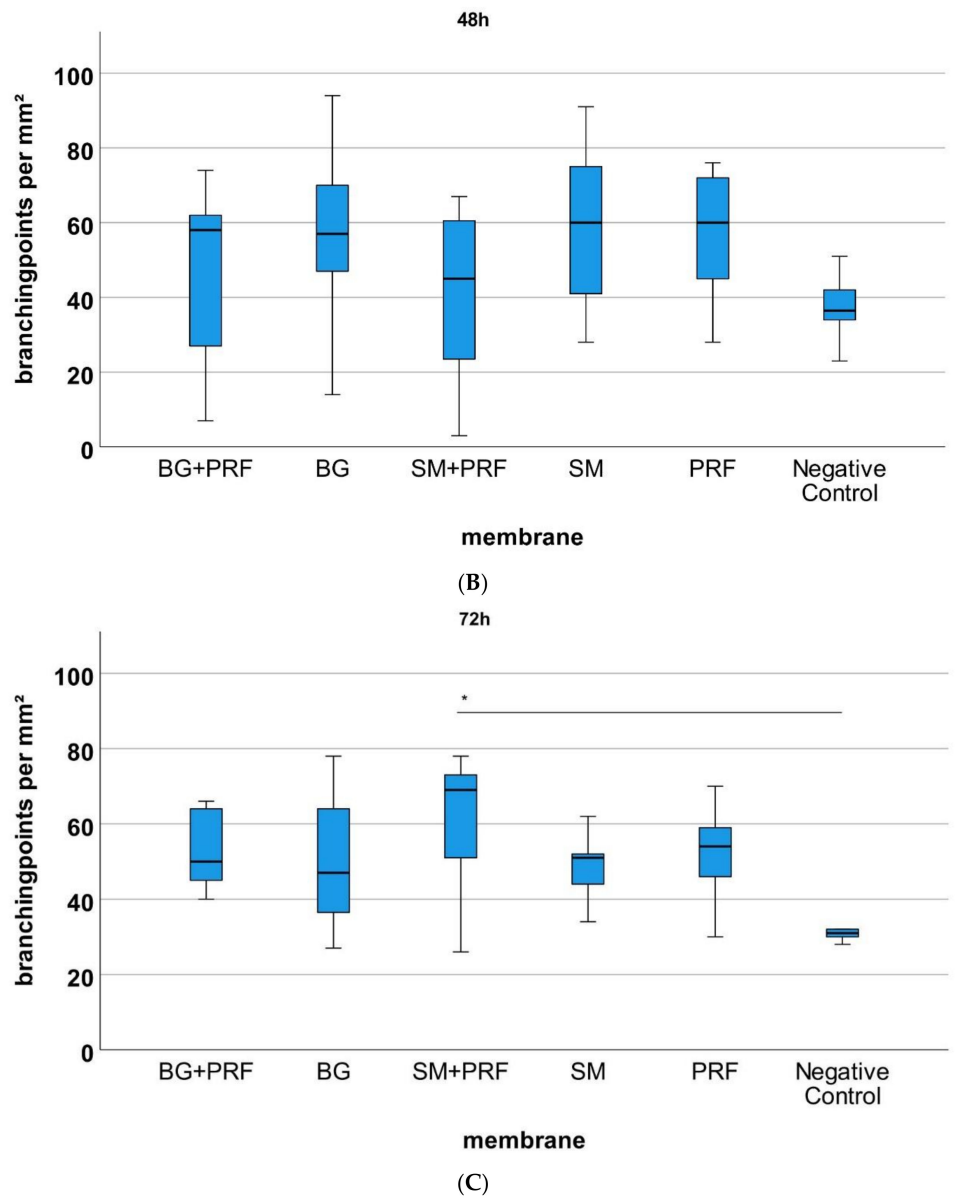
YSM assay and analysis of the branching points per mm^2^ for the respective matrix (BG + PRF: Bio-Gide^®^ with PRF, BG: native Bio-Gide^®^, SM + PRF: Symbios^®^ with PRF, SM: native Symbios^®^, PRF, negative control). (**A**) 24 h, (**B**) 48 h, (**C**) 72 h of incubation. * marks statistically significant differences (*p* < 0.05; for details please see the responding paragraph).

Comparing all groups (native vs. CM in combination with PRF vs. PRF alone vs. YSM alone) microscopically with each other, the statistical analysis of vessels (Figure 5) and branching points (Figure 6) per mm² showed no significant differences at 24 and 48 h after incubation (each *p* > 0.05). After 72 h, CM in combination with PRF induced statistically significant more vessels and branching points per mm² in comparison to native YSM (*p* < 0.01) but not vs. their native pendants (*p* > 0.05). However, there was a not significantly but descriptive increase in vessels and branching points per mm² in time for the bovine-derived membrane in combination with PRF. For PRF alone, however, a significant increase in vessels and branching points per mm² between 24 and 48 h (each *p* < 0.05) and between 24 and 72 h (each *p* < 0.05) was shown.

### 3.3. Immunohistochemically Analysis

**Figure 7:**
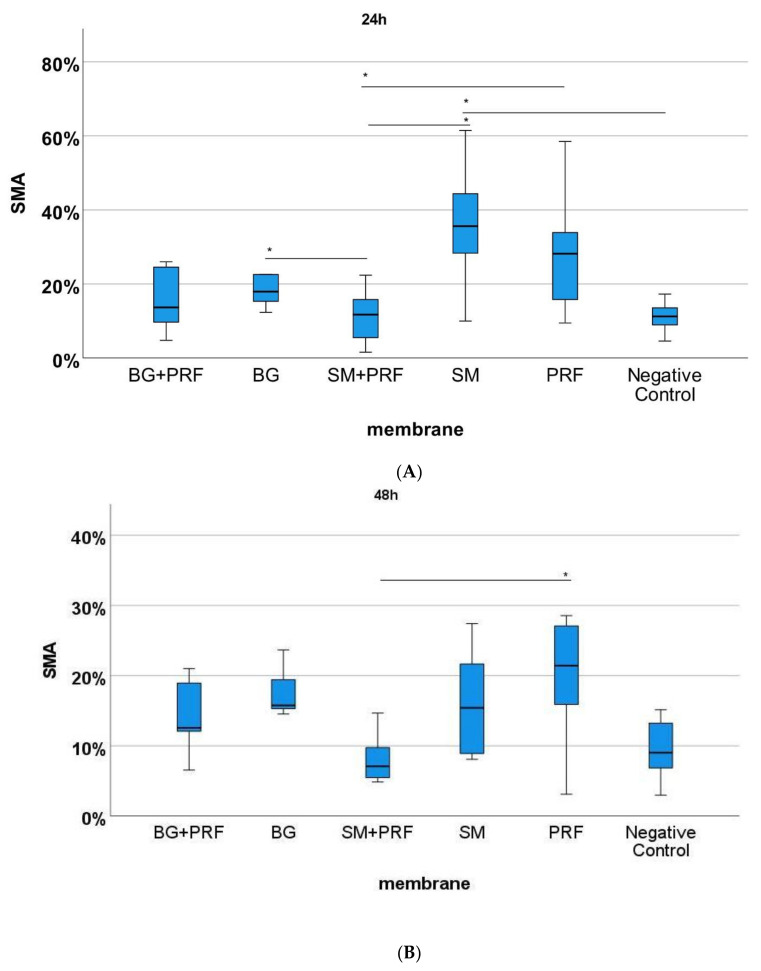

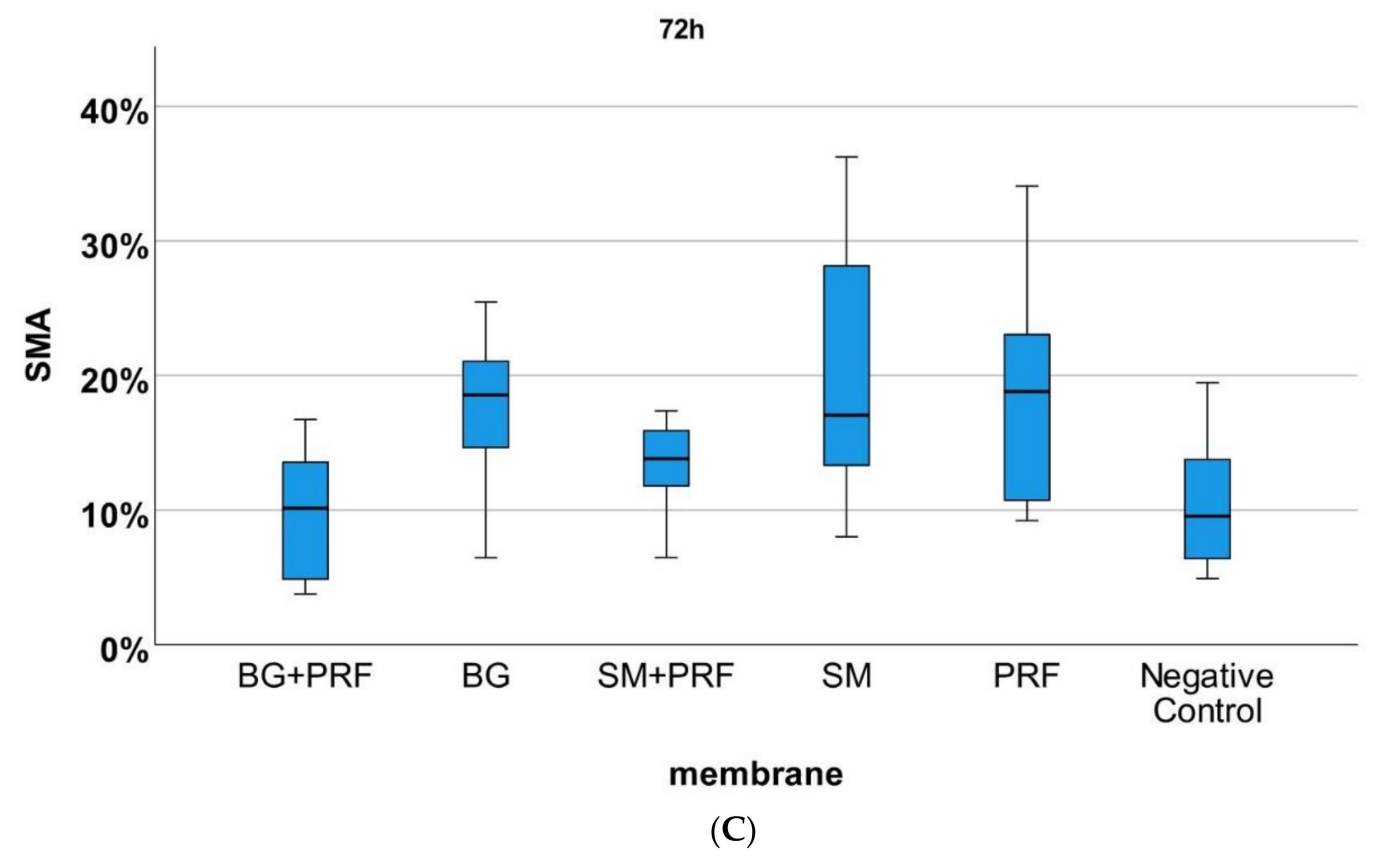
Analysis of aSMA immunohistochemical staining for the respective matrix (BG + PRF: Bio-Gide^®^ with PRF, BG: native Bio-Gide^®^, SM + PRF: Symbios^®^ with PRF, SM: native Symbios^®^, PRF, negative control). (**A**) 24 h, (**B**) 48 h, (**C**) 72 h of incubation. * marks statistically significant differences (*p* < 0.05; for details, please see the responding paragraph).

**Figure 8:**
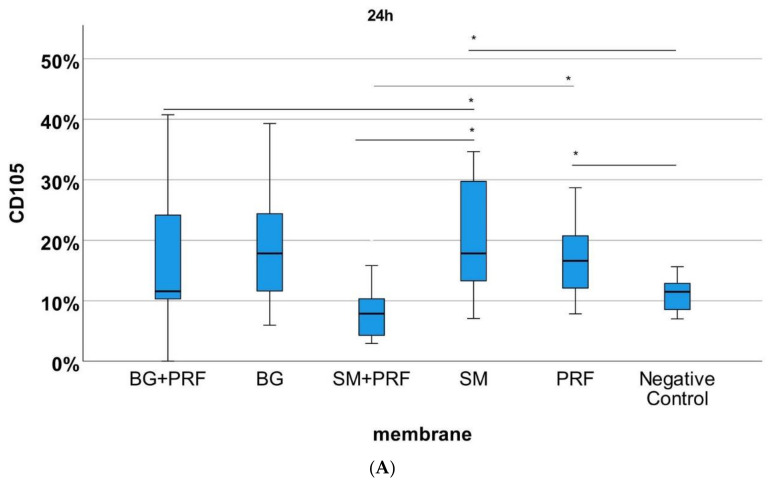

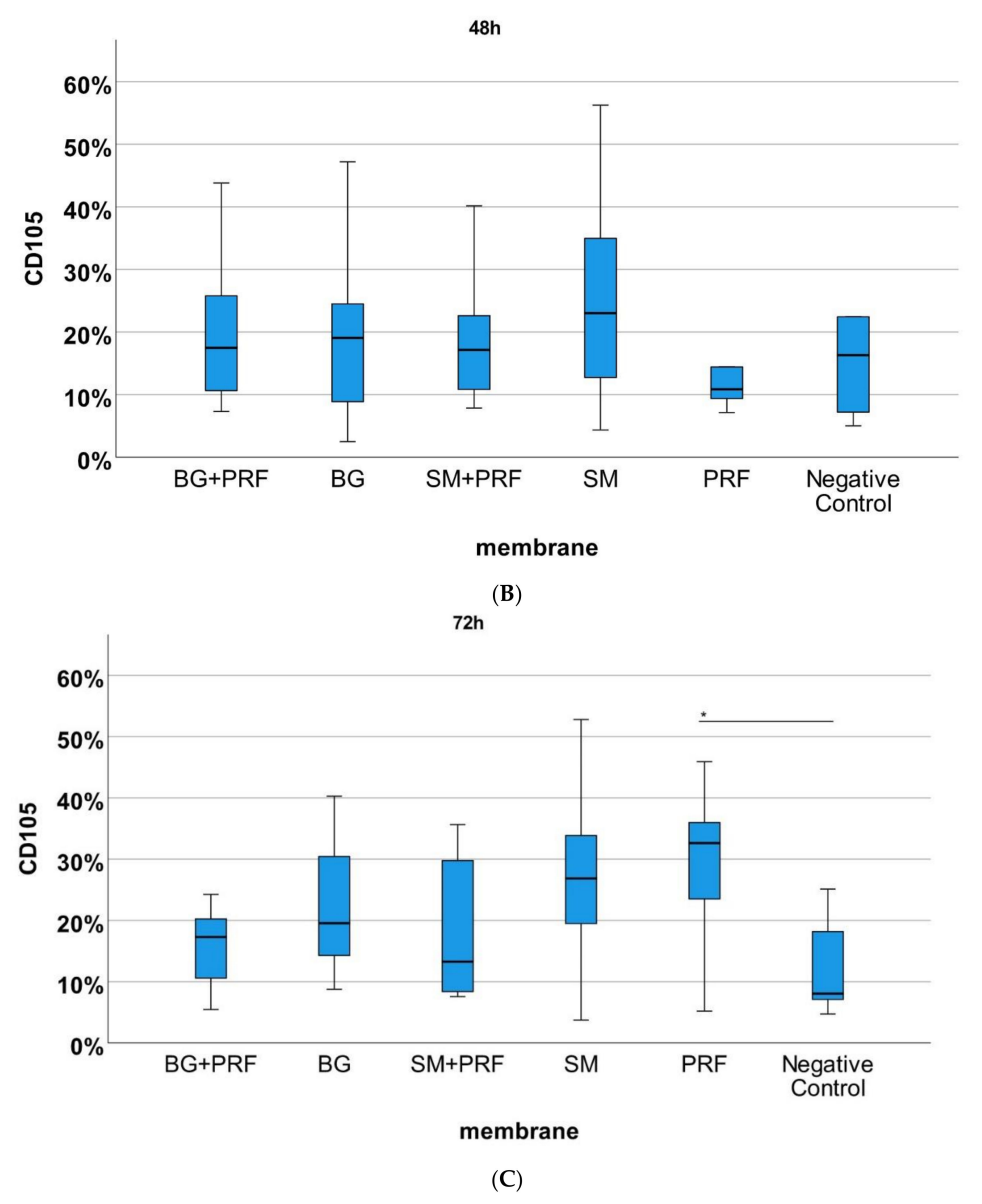
Analysis of CD105 immunohistochemical staining for the respective matrix (BG + PRF: Bio-Gide^®^ with PRF, BG: native Bio-Gide^®^, SM + PRF: Symbios^®^ with PRF, SM: native Symbios^®^, PRF, negative control). (**A**) 24 h, (**B**) 48 h, (**C**) 72 h of incubation. * marks statistically significant differences (*p* < 0.05; for details, please see the responding paragraph).

The immunohistochemically stained specimens (Figure 7 and Figure 8) showed for PRF alone compared with native YSM within 24 h a statistically significant increase in vessels in HE, αSMA and CD105 (each *p* < 0.05). After 72 h, statistically more vessels were found for PRF alone in comparison to native YSM in CD105 staining (*p* < 0.05). Compared with native YSM, a significant increase in vessel formation was found for native bovine CM in CD105 (*p* < 0.01) and αSMA staining (*p* < 0.05) after 24h and for bovine CM in combination with PRF (αSMA and HE staining between 24h and 72h, each *p* < 0.05). Native porcine CM demonstrated an increased number of vessels in αSMA staining compared with bovine CM in combination with PRF (*p* < 0.01) after 24 h. Native bovine CM showed higher number of vessels in CD105 staining compared with porcine CM in combination with PRF (*p* < 0.01). A statistically significant increase was also found in αSMA staining (*p* < 0.05) and CD105 staining (*p* < 0.01) after 24 h when PRF was compared with bovine CM in combination with PRF and in comparison with PRF and the combination of bovine-derived CM and PRF in CD105 after 48 h (*p* < 0.05)

## 4. Discussion

In this study, the impact of PRF for rehydration process of different xenogeneic collagen membranes as well as possible pro-angiogenic effects of the combination of different CM with PRF were analyzed. As a major result, significantly increased pH values were found at all points in time in PRF alone and its combined variants with Bio-Gide^®^ and Symbios^®^, compared with pure native saline solution and pH 7 solution. However, a significant trend to acidity in the observation time after 20 min was seen for PRF and its combined variants. Furthermore, PRF alone significantly increased vessel numbers and branching points in the YSM. After 72 h, CM in combination with PRF induced statistically significant more vessels and branching points compared with native YSM, but not vs. its native pendants. The immunohistochemical staining confirmed the results showing for PRF alone compared with native yolk sac membrane within 24 h a statistically significant increase in vessels. In context of wound healing, the pH value of the skin has also a central role, depending on the test localization and age pH values range from 4 to 6 [29]. In addition to vascular analysis, the present study aims to investigate changes in pH values due to the rehydration process of CM into different rehydration media. The pH value is defined as a measure of degree to which a solution is acidic or alkaline (scale ranges between 0 to 14). In blood, the pH values can vary from 7.35 to 7.45 [30]. A study by Nagaraja et al. investigated the effect of pH value changes on wound healing when liquid PRF was applied. Initially alkaline, it changed pH value to acidic on day 5. Thus, PRF may contribute to epithelialization and angiogenesis and promote wound healing [31]. However, PRF as matrix showed alkaline behavior and increased in alkalinity over 5 days. It can be concluded that it is best used for suppurative wounds, where the pH value is significantly acidic and PRF matrices can increase the pH value and produce normal levels [32]. In one review, positive wound healing associated with the use of PRF was found in 58% of the studies examined [33]. This is consistent with studies demonstrating an antimicrobial effect of PRF against bacteria in anaerobic milieus such as the dental root canal [34] or a local pyoderma gangrenosum [35]. This highlights the capacity of PRF to alter pH values even under anaerobic conditions [31]. In line with these results, the present study found the average pH value of PRF matrices did start at alkaline level but display acidity over time. The addition of Bio-Gide^®^ or Symbios^®^ CM did increase this effect. However, considering the different initial values in the media in the present study it is not surprising that there are significant differences in the comparisons between all groups. It must be pointed out that the saline solution did show significant lower pH values especially in combination with the respective CM in comparison to PRF with or without the addition of the CM. Since saline solution is regularly used in the clinical set up for rehydration protocols this is of direct clinical interest. Following above mentioned hypothesis of the influence of PRF to optimize pH values at wound healing sites, PRF seems more suitable for rehydration protocols of tested CM. Here, 5 min of rehydration seems feasible to avoid a subsequent decrease in pH values. In this context, CM of porcine and bovine origin are acellular and avascular compared with autologous scaffolds due to its multistage manufacturing process. Therefore, biofunctionalization by pro-angiogenic factors could be suitable to overcome this limitation. Proangiogenic effects of PRF or platelet concentrates could be based on growth factor release, such as VEGF, PDGF and FGF [10,36]. VEGF could induce endothelial cell proliferation and migration and the differentiation of precursor to mature endothelial cells. Next, progenitor cell stimulation could induce CM neovascularization. A stimulation of pre-existing vessels of the surrounding tissues could be possible, resulting in an increased sprouting and intussusceptive angiogenesis, and therefore an enhanced CM vascularization. On the other hand, an improved vascularization of CM and therefore VEGF release could even be associated with an improved bone remodeling since VEGF can increase endothelial cell activities and indirectly stimulate osteogenesis [37]. It can even be discussed whether an accelerated and improved vascularization of CM is without limitations. In comparison to others [38], the tested CM are not used as alternatives to oral soft tissue grafts from the palate for soft tissue regeneration, such as the coverage of periodontal recessions. These membranes were developed to cover and stabilize bone grafts, e.g., in the context of GBR. Next, these membranes could separate the bone grafts from the surrounding tissues and therefore delay soft tissue ingrowth to the bone grafts. This effect could be reduced by an accelerated vascularization, and therefore a fastened bio-degeneration. In this study, an already established model of chick embryo yolk sac membrane assay was used to examine the influence of platelet-rich fibrin, collagen membranes and its biologized variants on vascularization to accelerate and improve wound healing [39]. From some authors, the assay is seen as a possible bioreactor to culture and study the regeneration of human living bone [40]. Due to the high vessel density, the model is well suited for studying angiogenesis, which some current studies are taking advantage of. In particular, the effect of PRF seems to be very interesting. As in a previous study by our research group, a positive effect of PRF on angiogenesis was shown [22,41]. Ratajczak et al. could also improve the increase in vessels with PRF treatment using a similar assay, namely the chorion allantois membrane model (which consists of the same egg model in a later development state) [42]. The results are consistent with the present results: PRF alone significantly increased vessel numbers and branching points in the YSM. However, it must be admitted that due to the different PRF preparation protocols in different studies, a clear comparison is difficult [24]. In reconstructive surgery, artificial biomaterials such as bone substitutes or collagen membranes are used as a valid alternative to the autologous gold standard. However, with the wide range of offered products, it is often difficult finding the optimal scaffolding material. CM, such as Bio-Gide^®^ and Symbios^®^, the first one porcine-derived and the second one bovine-derived CM, represent such tools for regeneration techniques. One limitation of these procedures is the insufficient vascularization of the biomaterials, which makes integration into the recipient organism difficult and can cause possible immune reactions [32,43,44]. The innate and acquired immune system plays a decisive role in this [45]. In a study by Al-Maawi et al. Symbios^®^ CM were implanted subcutaneously in Wistar rats, to examine the tissue wound healing. After 30 days multinucleated giant cells were found on the matrix’s surfaces of the test group whereas only mononuclear cells and no vessels were found within the central region of the membranes. In the control group there were no multinucleated giant cells found, so that a foreign body reaction could be assumed in the test group. The effect of liquid PRF on CM was also investigated ex vivo. It was able to penetrate the membranes after 15 min. The study critically questioned the role of biomaterials and whether the cells are desirable in the context of vascularization or should be considered pathological [46]. Autologous platelet concentrates, such as the second generation of platelet PRF preparations, represent a “clinical” possibility for the pre-vascularization and functionalization of biomaterials [22,41,47]. Furthermore, the anti-inflammatory activity of PRF may support wound healing [48]. CM are used in clinical routine to enhance periodontal regeneration. However, neither various animal studies nor human clinical trials could show a complete regeneration [3]. In our study biologized variants of the membranes could achieve a descriptive increase in vessels and branching points in time but no advantage or disadvantage of Bio-Gide^®^ or Symbios^®^ CM could be found. The results could be explained with similar findings by another study of Al-Maawi et al. were only partial or superficial invasion of liquid PRF into the porcine CM was found [49]. On the other hand, in the presented results, both CM retained microscopically their shape and surface structure for 72 h, showed biocompatibility and its benefit in stability in comparison to PRF which is in accordance with other studies on biodegradation pattern of CM [38]. This is the novelty of the presented work in comparison to others: later time points could be observed that were not measured in earlier studies [22]. In addition, effect of PRF for rehydration process was scientifically assessed for the first time. In summary, further studies need to be carried out, to explore the full mechanisms, especially in biofunctionalization of membranes. This study suffers from some major limitations: First, one may question if the small number of experiments seems representative enough to reflect the complexity of the model and therefore, results should be interpreted with caution. As for the rehydration experiments, the experiment was not conducted with a bicarbonate buffer with the exact specification of the blood buffer to determine and analyze the activity of the bicarbonate buffer present in the blood serum. Furthermore, although an approved in vivo model was applied, the CAM assay has some errors such as uncontrollable external factors (transport and storage as well as incubation of the eggs, outside temperatures and different sizes of the eggs) that may have affected the values. Due to the individual characteristics of the eggs (size, shape, formation of the vessels, and movement of the embryo), the removal of the membranes could not be completely standardized. Thus, some of the membrane samples were not of the same size. Furthermore, the staining (technique?) is a limitation. The different sections are not fully comparable with each other due to their different gating. Lastly, the evaluation of the branching points and the number of vessels was carried out manually. In addition, vessel growth could only be assessed superficially and not in depth or underneath the matrix in one plane and one dimension, nor in their three-dimensional shape with the applied method. Overall, within the named limitations of the study, the presented study provides evidence for the use of autologous platelet concentrates in tissue regeneration procedures without the claim to fully reflect the whole complexity of angiogenic processes. 

## 5. Conclusions

In summary, this study could contribute to optimize future treatment concepts and implement the biofunctionalization of collagen membranes with PRF. However, the limitations of this study mentioned above (mainly small number of experiments, external/internal factors of CAM, superficial manual vessel counting, staining technique) must be considered. Furthermore, the illustrated in vivo effects are just a small aspect of the real effects in wound healing and tissue regeneration in humans. Further standardized clinical studies needs to be carried out, e.g., regarding the immigration and activation of granulocytes and how exactly PRF plays its role in vascularization and wound healing [50]. Therefore, the role of the pH value in context to wound healing and the application of different forms of PRF in acute or chronic wounds has also to be illuminated in detail. Here, future research could also include additive manufacturing of biocompatible scaffolds in combination with PRF, further improving bioengineering properties in cranio-maxillofacial surgery.

## Data Availability

Not applicable.

## Abbreviations

°C	degree Celsius
%	percent
3D	three-dimensional
α-SMA	anti-alpha smooth muscle Actin antibody (1A4)
µm	micrometer
BM	Bio-Gide^®^
CD105	Immunofluorescence anti-chicken antibody
CM	Collagen membranes
cm	centimeter
cm^2^	square centimeters
Dako	marked polymer-HRP anti mouse
GBR/GTR	guided bone- and tissue regeneration
h	hours
HE	Hematoxylin-eosin
min	minute
mL	milliliter
mm	millimeter
mm²	square millimeter
*n*	sample number
*p*	likelihood
PBS	phosphate buffered salt solution
PC	platelet concentrates
ph	pH value
PRF	platelet-rich-fibrin
s	second
SD	standard deviation
SM	Symbios Matrix^®^
VEGF	vascular endothelial growth factor
YSM	yolk-sac-membrane
